# Exposures to near-to-maximal speed running bouts during different turnarounds in elite football: association with match hamstring injuries

**DOI:** 10.5114/biolsport.2023.125595

**Published:** 2023-03-06

**Authors:** Martin Buchheit, Maxime Settembre, Karim Hader, Derek McHugh

**Affiliations:** 1 Kitman Labs, Dublin, Ireland; 2 Lille OSC, Lille, France; 3 HIITscience, Revelstoke, Canada

**Keywords:** Hamstring injuries, Planning, Programming, Football, Sprinting, Exposure

## Abstract

To describe the occurrence of near-to-maximal sprinting speed (near-to-MSS) running bouts during training and hamstring injuries during the consecutive match of the same turnaround in elite football (soccer). Retrospective data from 36 team-seasons (16 elite teams performing in top European leagues) were analyzed (627 players, 96 non-contact time loss match hamstring injuries). We described 1) the occurrence of > 85%, > 90% or > 95% MSS exposures during training within each turnaround and match hamstring injuries and 2) whether the above-mentioned injury occurrences differed depending on the day(s) of the turnarounds (i.e., the period separating two consecutive matches, which is generally from 3 to 8 days) when these speed exposures occurred. The longer the length of the turnarounds and the lower the speed thresholds, the greater the number (and proportion) of near-to-MSS exposures (e.g., 18%, 45% and 72% of turnarounds with > 85% runs for 3, 5 and 7-turnarounds, respectively). For half of the turnarounds examined, there were no match hamstring injuries when players were exposed to running bouts > 95% MSS during training (e.g., injury rates: 0; CI: 0–15). Injuries still occurred during 85% of the turnarounds when there were no or lower relative speed exposures (i.e., > 85 or > 90%, injury rates: 2–5, CI: 0–6). Finally, irrespective of the turnaround length, there were no match hamstring injuries when > 95% MSS exposures occurred at D-2, while in contrast, injuries still happened when players were not exposed at all, or when these exposures occurred at D-3 and/or earlier within the turnaround. While the present observational study design precludes the examination of causal relationships, the programming of > 95% MSS exposures at D-2 may help mitigate match hamstring injury occurrences in elite football.

## INTRODUCTION

Hamstring strain injuries remain the most prevalent time-loss injuries in professional football [1]. While their relative occurrence may have slightly decreased in relation to the likely increased match demands over the past decade [2], practitioners are still seeking mitigation strategies both in the gym and on the pitch [3]. Among the different recommended strategies, the use of eccentric-based exercises [4] and exposures to near-to-maximal sprinting speed (near-to-MSS) running bouts (either with or without the ball) are now the most recommended [5, 6]. Sprinting is indeed both complex and unique at many levels (e.g., legs interaction, elastic energy transfer, reflexes, kinematics, kinetics) [6] and a similar recruitment intensity of the hamstring muscles (i.e., electromyographic activity) cannot be reached with isolated gym exercises [7].

In practice, recent studies have shown relationships between hamstring strain injuries and near-to-MSS exposures both in Australian Rules Football [8, 9] and Gaelic Football [10] players. More precisely, both under and over near-to-MSS exposures were associated with higher injury rates, suggesting the existence of an optimal chronic “dose” i.e., a number of weekly exposures [8, 10] and/or monthly cumulative distance [9]. This optimal chronic dose is likely specific to each population and context, and it is therefore difficult to provide guidelines for all practitioners on the back of those two studies. More importantly, those studies do not provide clear guidelines on how and when to program these near-to-MSS exposures during turnarounds of different lengths. A turnaround refers to the period separating two consecutive matches, which is generally from 3 days (2 full days of recovery and the day of the second match, such as when playing on Sunday and then again on Wednesday) to 8 days (7 days in between matches, such as playing on a Saturday and then the next Sunday). How fast football players should run is also still unclear, since large variations in relative velocities have been reported, ranging from ≥ 80 [9], to 85 [8] or even 95% of MSS [10].

In fact, the questions of 1) the optimal intensity (in % of MSS) and 2) on which day to program these near-to-MSS exposures have not been examined scientifically despite their immense importance in terms of match performance and hamstring injury management [11]. The only partial answer to this question that is available to us today comes from the 100 elite football (soccer) practitioners that we surveyed in 2021 [12]. While the large majority of the responders confirmed the need to regularly expose players to these high-speed running bouts, there was a lack of agreement as to when MSS work should be programmed, especially whether it should occur 2 or 3 days before the match i.e., D-3 vs D-2. This was likely due to the lack of robust evidence, and this programming practice was instead based on experience and/or adherence to typical periodization paradigms and models (e.g., tactical periodization [13], R. Verheijen [14], or el modelo estructurado of FC Barcelona [15]).

In order to shed light on this important topic, we retrospectively analyzed data from 19 elite teams performing in top football leagues across the globe. The first aim of the study was to describe the occurrence of both match hamstring injuries and > 85%, > 90% or > 95% MSS exposures during training within different turnaround lengths. The second aim was to examine match hamstring injury occurrences as a function of the day(s) of the turnarounds when these speed exposures occurred.

## MATERIALS AND METHODS

### Study design and procedures

**FIG. 1:**
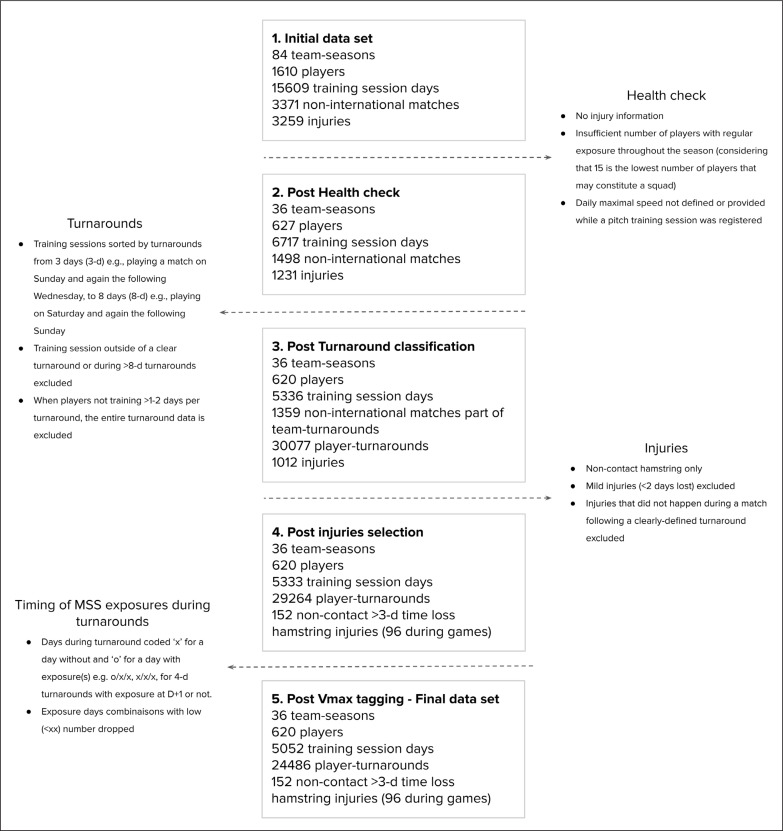
Flow chart showing the data selection process.

The overall research was based on retrospective analyses of both match hamstring injury occurrences and players’ training locomotor (running) activities collected via an online database (i.e., Kitman Labs platform, Dublin, Ireland) commonly used by all the football (soccer) teams involved in the study. Figure 1 shows the flow chart of the data selection process.

### Population

The elite adult football players from whom data was examined belonged to 19 different teams competing in the EPL, the Italian Serie A, the French Ligue 1, the Bundesliga, the Scottish Premiership, the MLS and the Dutch Eredivisie (from January 2018 to December 2021). This initial sample represented 84 team-seasons (Figure 1).

Then various exclusion criteria were applied at the season level:

1. those with no injury information
2. an insufficient number of players with regular exposure throughout the season (considering that 15 is the lowest number of players that may constitute a squad)
3. daily maximal speed not defined or provided while a pitch training session was registered
4. After applying those exclusion criteria the final sample represented 36 team-seasons.

### Data extraction and anonymity

Each player and club is provided with an ID number on the platform. The researchers in charge of the analysis could only pull and analyze data associated with their IDs – no names included. Then, data was transformed and coded for injury occurrence (dates only used for assessing occurrences, such as during a match vs. during training and when in relation from/to the previous match) and type (contact or non-contact injury, without any more details), to provide a final dataset. The medical staff of each team registers injury details in the platform as a part of their daily player care management, including variables such as date of injury, type and location of the injury, as well as severity (days lost). Similarly, players’ match and training session participation are recorded as part of the team staff’s daily monitoring. Additionally, the measures of training and competitive load are also added to the platform. The fact that all clubs used the same platform ensured the standardisation and the reliability of all types of entries, from medical information to exposure measures (e.g., session duration and GPS data attached to the system calendar). We nevertheless ran a thorough data health check to ensure that all data retained for analysis met the same standard. In addition to all the steps above that guaranteed high levels of both data security and anonymity (https://www.kitmanlabs.com/privacy-security-and-compliance/), permission was granted by the teams for their inclusion in this research study, therefore ethics committee clearance was not required [16].

### Turnarounds

A *n*-d turnaround was defined as a microcycle with *n* days between the first and second match, where *n* is the count of days from the first day after a match up to and including the following match day. The shortest observed turnaround was 3 days (3-d) e.g., playing a match on Sunday and again the following Wednesday, while the longest was 8 days (8-d) e.g., playing on Saturday and again the following Sunday. The longer and less common turnarounds (e.g., ≥ 9 days, likely including international breaks or holidays, when the training dynamics are completely different than during typical in-season turnarounds) were excluded from the analysis (Figure 1).

### Injuries and turnaround participation

In this study we focused on non-contact hamstring injuries (i.e., a substantial strain of either the biceps femoris, semitendinosus, or semimembranosus muscle), as registered by the medical staff of each club, using the Orchard Sports Injury and Illness Classification System (OSIICS) offered by the online platform. While the exact diagnostic methods are impossible to describe in detail given the large variability of staff involved (i.e., 19 teams with likely more than 25 to 30 practitioners in total), the large majority of teams (if not all) at the elite football level have access to high-quality scans (i.e., Echography, Magnetic Resonance Imaging). In the literature, an injury is often defined as an occurrence sustained during either training or match-play which prevents a player from taking part in training or match-play for 1 or more days following the occurrence [17]. In this study, in contrast, we wanted to focus on hamstring injuries that substantially impact training and match participation; so we only considered injuries that caused a minimum of 3 days of training/playing interruption i.e., ≥ 3-day time loss. In fact, we excluded all mild injuries (< 2 days lost) because injuries in this category could conceivably not have an impact on the next game availability or training dynamic within the same turnaround. In addition, this choice has allowed us to exclude non-substantial injuries that may have resulted in a few days of unavailability due to potential training removal, as it sometimes happens in clubs (i.e., this refers to load management, when players are not injured but taken out of training for precaution – which generally allows them to train fully the next day). If the medical staff registered injuries from the start to the end of the season, we assumed that they strictly adhered to this practice throughout the whole season and that there was no missing data for this metric in this situation.

Only the data of players who played the match that ended the current turnaround were used for analysis, and that was considered as a ‘player-turnaround’. Player-turnarounds in which any injuries occurred, other than non-contact time loss hamstring injuries, were removed from the analysis (Figure 1).

### Near-to-maximal sprinting speed exposures

The maximal sprinting speed of a player was calculated based on the available data. The ideal scenario was when a club was actually testing for MSS, and in this case, the resulting MSS (collected by GPS) was used for analysis. When proper testing data was not available, we used the average of the three highest speeds reached in the entire GPS data set of each player (after having manually removed all possible erroneous data > 37 km/h) [11]. This later approach (i.e., using the max speed reached by a player in any possible condition measured by a GPS) is actually how people work in clubs – we therefore believe that, despite the possible underestimation of MSS for some players (which will be never known), it makes the results more ecological and reflect well the real-world scenarios. Following both the research literature [8–10] and actual sports science practice in the field, we marked when near-to-MSS exposures occurred using individual speed thresholds at > 85%, > 90% and > 95% of each player’s MSS [11].

### Near-to-MSS exposures during turnarounds

To understand the frequency of near-to-MSS events, we labelled all individual player training sequences leading to a match by the turnover value and added an indicator as to whether a near-to-MSS event had occurred or not. We coded the entire individual player training sequences leading to the match (as a block of 2 to 7 days for 3- to 8-d turnarounds) as including (true) and not (false) one or more near-to-MSS exposure using > 85%, > 90% and > 95% of each player’s MSS [11]. While other speed thresholds could have been used (e.g., 80% MSS), we replicated those used in a previous study on elite football players using the exact same approach [11]. Moreover, using > 85%, > 90% and > 95% reflects very well the common practices in elite football (Figure 1).

### Timing of near-to-MSS exposures during the turnarounds

The timing of near-to-MSS exposures was analyzed in two ways. First, to understand the actual programming of near-to-MSS exposures, the individual player near-to-MSS exposure distribution patterns were coded within each microcycle. Each day was labelled as to whether a near-to-MSS exposure occurred, say ‘x’ for a day without and ‘o’ for a day with exposure(s), and the frequency of each of the possible combinations e.g. x/x/x, o/x/x, x/o/x, x/x/o, o/o/x, o/x/o, x/o/o, o/o/o for 4-d turnarounds assessed for each turnaround. Second, since coaches generally split the between-match training cycle into two phases (recovery/compensation and intense work from D+1 until D+3, and match preparation from D-2 to D-1), we also grouped together the first training days of each turnaround up to and including D-3 e.g., for a 7-d turnaround we grouped D-6 to D-3 together as D-3, and D-2 and D-1 were considered as unique days. This grouping allowed us to approximate all turnarounds as a standard 3-day turnaround. Since 3-d turnarounds do not include a D-3, this microcycle was only included in a part of this analysis (Figure 1).

### Statistical analysis

Considering all the inclusion/exclusion criteria above, the final analysis was run on a total of 620 players participating in 5052 training session days and 1358 non-international matches for a total of 24486 player-turnarounds (3 to 8 days), and 152 hamstring injuries, with 96 of those occurring during matches, as part of the 36 team-seasons (Figure 1).

Since preliminary analysis did not show any trends suggestive of differences between the different leagues or continents, all data were pooled together to increase the sample size.

Injury rates were estimated as follows: number of non-contact time-loss match injuries following a specific training combination for a given turnaround length/number of observations of that specific training combination for this particular turnaround length × 1000 (e.g., number of match injuries following the o/x/x combinations / the number of o/x/x combinations observed × 1000, or (6/900) × 1000 = 6.06). Results are presented as a mean and 95% confidence intervals (using the exact binomial approach) [18]. Substantial differences were assumed when the CIs did not overlap [19].

## RESULTS

### Near-to-MSS running bouts occurrences

The number of player-sequences within each turnaround examined where > 85%, > 90% and > 95% MSS exposures occurred (true) or not (false) during the training session days leading to the match are shown in Table 1, 2 and 3, respectively. Overall, the proportion of training sequences with at least one near-to-MSS exposure was clearly lower than without, i.e., 40%, 24%, 10% for > 85, > 90 and > 90% MSS, respectively.

**TABLE 1:** Number of player-sequences within each turnaround examined where > 85% MSS exposures occurred (true) or not (false) during the training session days leading to the match, number and rate (95% confidence limits, CL) of hamstring injuries during matches.

	Occurrence of > 85% MSS running bouts during the training session days leading to the match	Number of player- sequences	Number of hamstring match injuries	Injury rate/1000 sequences (95% CL)
3 d	False	7389	33	4.5 (2.9–6)
	True	1626	9	5.5 (1.9–9.1)
4 d	False	5663	17	3.0 (1.6–4.4)
	True	2750	9	3.3 (1.1–5.4)
5 d	False	1461	2	1.4 (0–3.3)
	True	1177	2	1.7 (0–4.1)
6 d	False	1037	1	1.0 (0–2.9)
	True	1580	4	2.5 (0.1–5)
7 d	False	1430	4	2.8 (0.1–5.5)
	True	3677	11	3.0 (1.2–4.8)
8 d	False	439	0	0.0 (0–8.4)
	True	1207	4	3.3 (0.1–6.6)
**Total**	False	17419	57	3.3 (2.4–4.1)
	True	12017	39	3.2 (2.2–4.3)

**TABLE 2:** Number of player-sequences within each turnaround examined where > 90% MSS exposures occurred (true) or not (false) during the training session days leading to the match, number and rate (95% confidence limits, CL) of hamstring injuries during matches.

	Occurrence of > 90% MSS running bouts during the training session days leading to the match	Number of player- sequences	Number of hamstring match injuries	Injury rate/1000 sequences (95% CL)
3 d	False	8259	38	4.6 (3.1–6.1)
	True	756	4	5.3 (0.1–10.5)
4 d	False	6933	22	3.2 (1.8–4.5)
	True	1480	4	2.7 (0.1–5.3)
5 d	False	1992	4	2 (0–4)
	True	646	0	0 (0–5.7)
6 d	False	1724	4	2.3 (0–4.6)
	True	893	1	1.1 (0–3.3)
7 d	False	2564	7	2.7 (0.7–4.7)
	True	2543	8	3.1 (1–5.3)
8 d	False	843	2	2.4 (0–5.7)
	True	803	2	2.5 (0–5.9)
**Total**	False	22315	77	3.5 (2.7–4.2)
	True	7121	19	2.7 (1.5–3.9)

**TABLE 3:** Number of player-sequences within each turnaround examined where > 95% MSS exposures occurred (true) or not (false) during the training session days leading to the match, number and rate (95% confidence limits, CL) of hamstring injuries during matches.

	Occurrence of > 95% MSS running bouts during the training session days leading to the match	Number of player-sequences	Number of hamstring match injuries	Injury rate/1000 sequences (95% CL)
3 d	False	8774	42	4.8 (3.3–6.2)
	True	241	0	0 (0.0–15.2)
4 d	False	7837	23	2.9 (1.7–4.1)
	True	576	3	5.2 (0–11.1)
5 d	False	2416	4	1.7 (0–3.3)
	True	222	0	0 (0.0–16.2)
6 d	False	2254	5	2.2 (0.3–4.2)
	True	363	0	0 (0.0–10.1)
7 d	False	3902	12	3.1 (1.3–4.8)
	True	1205	3	2.5 (0–5.3)
8 d	False	1310	2	1.5 (0–3.6)
	True	336	2	6 (0–14.2)
**Total**	False	26493	88	3.3 (2.6–4)
	True	2943	8	2.7 (0.8–4.6)

**FIG. 2:**
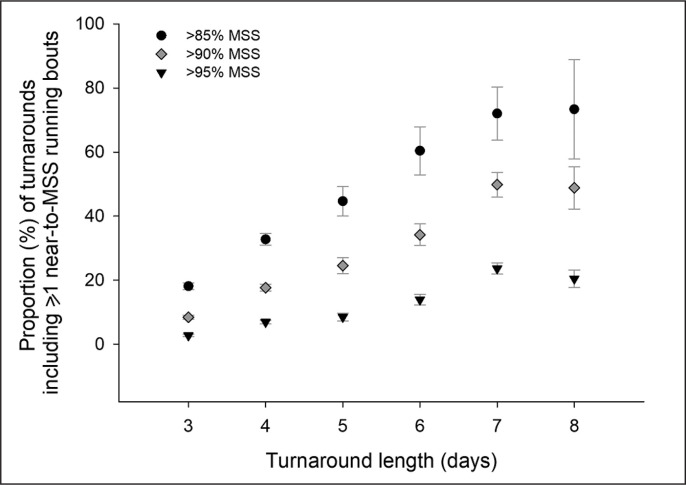
Proportion of (training) player-sequences including at least one > 85%, > 90% and > 95% MSS running bouts occurrence as a function of the length of the turnaround. Error bars represent 95% confidence intervals.

When looking at the turnaround level, the longer the length of the turnarounds and the lower the speed thresholds, the greater the number (and proportion) of near-to-MSS exposures (Figure 2 and Tables 1–3). For example, when considering > 85% MSS exposures, there were 2 (5-d turnarounds) to 5 × (3-d turnarounds) more sequences without exposures than with. For the longest turnarounds however, sequences with near-to-MSS exposures were 2 (7-d turnaround) to 3 × (8-d turnaround) greater than those without.

### Overall occurrence of near-to-MSS running bouts and match hamstring injuries

**FIG. 3:**
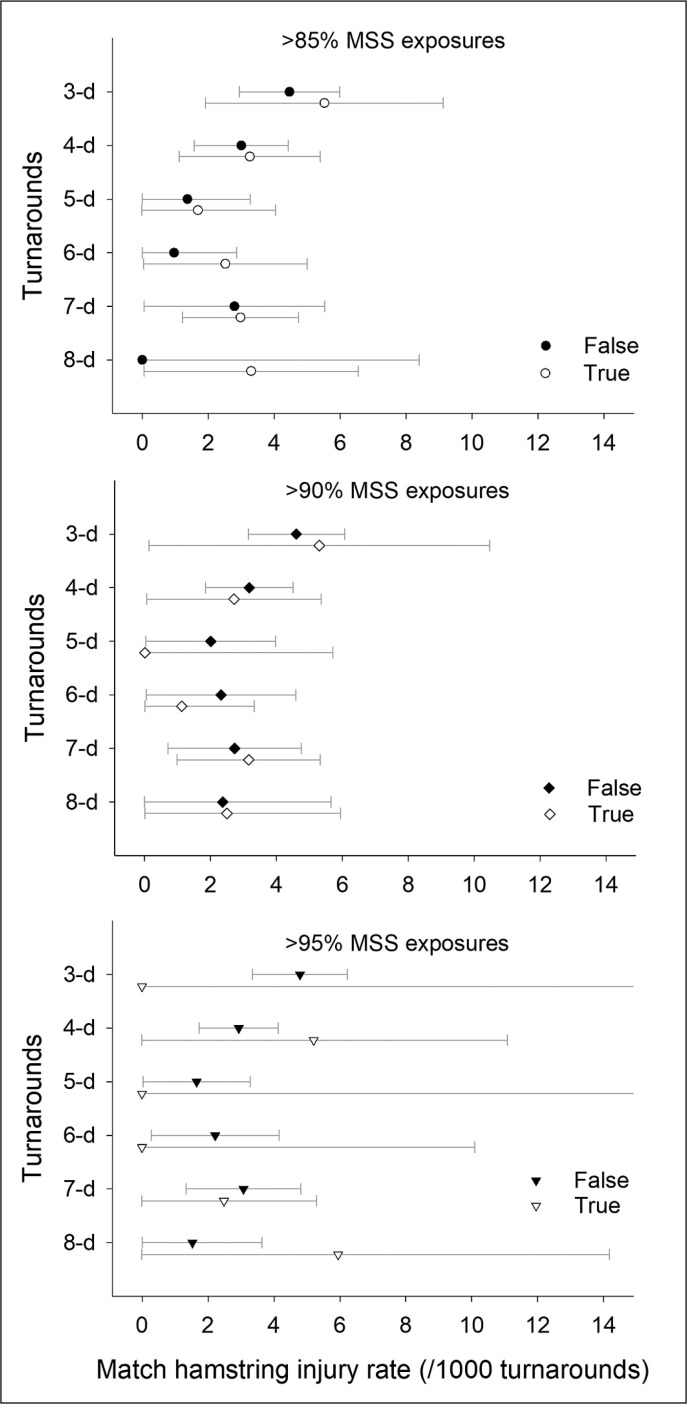
Match hamstring injury rate (with 95% confidence intervals, and per 1000 turnarounds participation) in players achieving (true) or not (false) > 85% (upper panel), > 90% (middle panel) or > 95% (lower panel) of their maximal sprinting speed (MSS) during the training session days leading to the match, for the different turnarounds examined.

When looking at all turnarounds pooled, there was no difference in injury rate between training sequences including vs. not including near-to-MSS running exposures, irrespective of the speed threshold considered (Table 1–3). However, when looking within each turnaround, there were no match hamstring injuries when players were exposed to running bouts > 90% MSS (i.e., 5-d turnaround) and > 95% MSS (i.e., 3-, 5- and 6-d turnaround) during the training sessions days leading to matches (Table 3 and Figure 3 lower panel).

In contrast, injury rate was still substantial when considering running bouts > 85%, and when looking at the majority of turnarounds with > 90% MSS exposures (Table 1 and 2, Figure 3 upper and middle panel).

### Daily programming of near-to-MSS exposures and match hamstring injuries during 4- to 8-d turnarounds pooled

When looking specifically at the day(s) when > 95% MSS was reached within an average turnaround (i.e., 4- to 8-d turnarounds pooled), there were four main patterns with large sample sizes (n > 200): near-to-MSS occurrence at D-3 and before, n = 990 player-turnarounds and 6 injuries; at D-2, n = 480 and 0 injuries; at D-1, n = 215 and 2 injuries, and no exposure throughout the turnaround, n = 11168 and 46 injuries. The other day-combinations (e.g., occurrences both at D-2 and D-1) had all very low sample sizes (n < 50), for a total of 126 player-turnarounds in total and no injuries; these later combinations were not used for analysis.

**FIG. 4:**
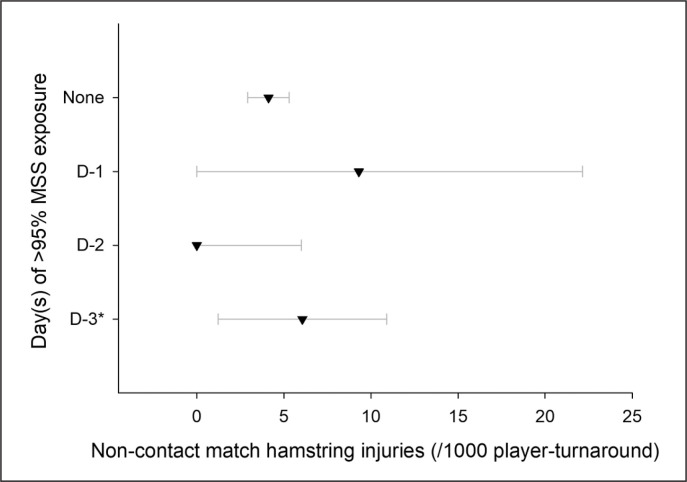
Match hamstring injury rate (with 95% confidence intervals, and per 1000 player-turnarounds) in relation to the training session day(s) of the turnaround when running bouts > 95% MSS occurred. *Note that D-3 is an aggregation of all training session days of the turnaround before D-3 included (e.g., D-3 summarizes occurrences from D-6 to D-3 for a 7-d turnaround, see methods). Data presented here are from 4- to 8-d turnarounds pooled together; since there is no D-3 data during 3-d turnarounds, data from the entire 3-d turnarounds is excluded from this analysis.

When examining the pooled data and comparing these main four patterns, there was no observation of match hamstring injury when > 95% MSS was reached at D-2 – and only for that day (Figure 4). In contrast, injuries still happened when players were not exposed at all, or when these exposures occurred at D-3 and/or earlier within the turnaround. The difference in injury rate between exposures at D-2 vs. D-1 was unclear, likely due to the very low number of injuries for the latter (n = 2).

### Daily programming of near-to-MSS exposures and match hamstring injuries during all turnarounds pooled

During 3-d turnarounds (excluded from the above analysis since not including D-3 data), 97% of the player-sequences (n = 5854 player-turnarounds and 42 injuries) did not include 95% MSS exposures, with a hamstring injury rate of 7.1 (6.1–8.3). The numbers of other player-sequences were all below 80, with no injury when > 95%MSS exposures occurred at D-2 or D-1.

**FIG. 5:**
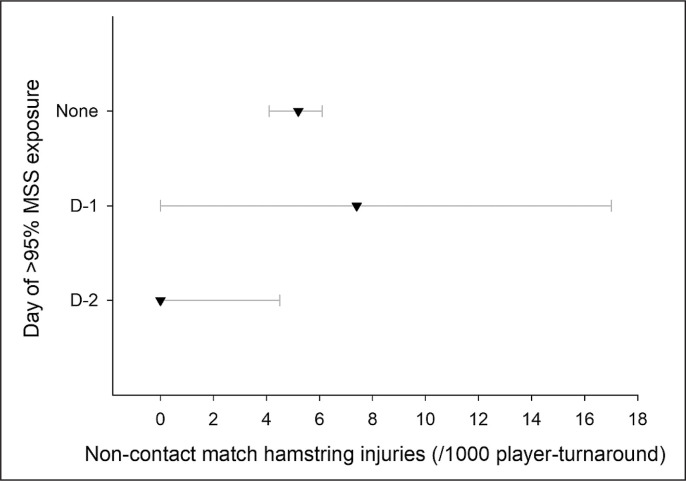
Match hamstring injury rate (with 95% confidence intervals, and per 1000 player-turnarounds) in relation to near-to-MSS exposure over the last two training day(s) of the turnaround – 3-d to 8-d turnarounds pooled.

When adding the 3-d turnarounds to the previous analysis to increase the number of injuries up to 96 in total (Figure 5, but then removing the D-3 aggregation to be consistent), the trends were similar to those in Figure 4, but there was almost no overlap anymore between the D-2 vs. non-exposure alternatives (Figure 5).

## DISCUSSION

This is to our knowledge the first study to describe the occurrence of both non-contact time loss match hamstring injuries and near-to-MSS running bouts within typical turnaround in elite football. The main findings were the following: 1) the large majority of players arrived at the match without having been exposed to near-to-MSS running bouts during the training days of the current turnaround (60% for > 85% MSS, 76% for > 90% MSS and 90% for > 95% MSS), 2) the longer the length of the turnarounds and the lower the speed thresholds, the greater the number (and proportion) of near-to-MSS exposures, 3) for half of the turnarounds examined, there were no match hamstring injuries when players were exposed to running bouts > 95% MSS during training, 4) injuries still occurred during 85% of the turnarounds when there were no or lower relative speed exposures (i.e., > 85 or > 90%) and finally, 5) there were no hamstring injuries when > 95% MSS exposures occurred at D-2, while in contrast, injuries still happened when players were not exposed at all, or when these exposures occurred at D-3 and/or earlier within the turnaround.

### Near-to-MSS running bouts occurrences

The most common practice was not to touch near-to-MSS running speeds during training. On average, there were 3 to 10 × more player-turnarounds without near-to-MSS exposures than with, and it was only during the longest turnarounds that these higher running speeds were reached (Figure 1).

The first part of these findings is not surprising and is likely related to the type of drills programmed by most coaches, which do not allow players to reach high speeds [20]. It is now well established that small-side games over small spaces are insufficient in this regard (since players may need to maximally sprint over at least 30 m to reach near-to-MSS speeds [21]), and that often, the only way to get players exposed to near-to-MSS exposures is to either program finishing and transition drills with enough depth [22] and/or individual sprinting drills with or without the ball [23]. The current results (discussed below) lend support to this latter practice.

The influence of the turnaround length on near-to-MSS running bout occurrence is also consistent with the results of our recent survey [12], where the most important drivers for the programming of almost all training contents, and especially those demanding either at the neuromuscular or metabolic level, were reported to be the distance from and to the next match. With not enough time between matches, the emphasis is put on recovery, and practitioners likely consider maximal sprint work too demanding to be performed close to the previous match (the residual fatigue from the previous match may increase injury risk during sprint training itself). In fact, during periods of match congestion, the typical training programming (recovery and easy sessions) does not allow near-to-MSS exposures for starters; those higher-speed exposures may only be possible (and required, see below) for substitutes. When to program those high-speed exposures for subs is another great question for practitioners, and a tentative answer to this will be provided in the last part of the discussion. Finally, these results are also consistent with the common trend found both in the scientific literature [24] and the coaching community, suggesting that 48 h of recovery is generally needed between sprint training sessions/events.

### How fast is enough?

While researchers have shown associations between high-speed exposures and injury risk [8–10], there was still a lack of evidence about the minimal intensity required for those runs to be protective. In fact, when it comes to selecting the minimal running velocity that may be associated with reduced hamstring injuries, large variations in relative velocities have been reported, ranging from ≥ 80 [9], to 85 [8] or even 95% of MSS [10]. While the present observational study design precludes the examination of causal relationships, our results show for the first time in a very large sample of elite football players (620 players for a total of 24486 player-turnarounds, Figure 1), that near-to-MSS exposures may need to be performed > 95% MSS during training to be associated with reduced match hamstring injuries (Figure 3). While limited with the present data, the fact that > 95% MSS exposures may be associated with lower injury rates than when only reaching lower relative speeds (85% and 90% MSS), may be related to both higher levels of movements specificity (e.g., leg interaction, elastic energy transfer, reflexes, kinematics, kinetics) [6] and hamstring muscles recruitment that increases exponentially with running speed. In fact, the eccentric (negative) work done at the knee appears to be related to the square of running velocity, which means that toward the attainment of maximal speed (> 95%) the work done is steeply ramping up in a disproportionate manner [25].

### Programming near-to-MSS running bouts during the training microcycle

Previous research had suggested the existence of an optimal chronic “dose” in terms of near-to-MSS exposures (i.e., number of weekly exposure [8–10] and/or monthly cumulative distance) [9]. However, this optimal chronic dose is likely specific to each population and context, and it is, therefore, difficult to provide guidelines for all practitioners on the back of those three studies. More importantly, those studies did not provide clear guidelines on how and when to program these near-to-MSS exposures during the weekly microcycle and during turnarounds of different lengths. For these reasons, we believe that our results shed some light on the potential (more) optimal practices in the field.

In this very large data set, there were no match hamstring injuries when near-to-MSS exposures were programmed at D-2. Importantly, this was the case only when near-to-MSS exposures were programmed on that day (Figures 3 and 4). Despite the overlap of the CIs, and acknowledging that descriptive information does not imply causality, this trend suggests that reaching near-to-MSS at D-2 may be the most advantageous strategy with respect to match hamstring injury occurrence.

The actual programming of MSS exposures at D-2 vs. D-3 was actually one of the most debated areas among the practitioners we surveyed [12]. In accordance with the discussion around the alternation of moderate vs. light loads between D-2 and D-1, the sequence order of high-speed running (HSR) and MSS work may have some relevance in the context of injury risk. In fact, since high training loads including HSR and playing over large spaces (which are mainly programmed on D-3, irrespective of the periodization approach [12]) likely induce acute posterior chain fatigue [26], the programming of MSS work the next day (D-2) could expose players to a higher risk of injury during those sprints (assuming that increased neuromuscular fatigue and the changes in mobility/pelvic control that follow such sessions increase injury risk) [27, 28]. For that reason, probably, and in somewhat contradiction with the orientation of the tactical periodization [13] approach that advises planning speed work on D-2 [12], 75% of practitioners reported to program MSS on the same day as HSR (D-3) for both 6- and 7-day turnovers (see Figure 7). This is often achieved during game-play sequences over large spaces [22] and/or through specific speed top-ups post-session when speed targets are not reached [23]. Albeit anecdotal, several practitioners commented in their notes that while they had started to program MSS work at D-2 in the line of the tactical periodization paradigm [13], they ended up changing this specific programming aspect for the above-mentioned reasons [29, 30]. Another important comment in relation to this specific point, is that having ‘speed’ as the focus of the third acquisition day (following ‘strength’ and ‘endurance’ [12] have been sometimes misunderstood: ‘speed’, as originally introduced, may not necessarily involve MSS work, but could simply refer to the speed of execution, which is often implemented via short attacking transition work and finishing actions.

While the benefit of programming vs. not programming near-to-MSS exposure is straightforward (i.e., preparing muscles to match-specific demands) [5, 6], it remains unclear why exposing players at D-2 may be more appropriate than at D-1 or D-3 and earlier (if this is that clear, considering the CIs overlaps, though). This may be related to the recovery time course of the posterior chain muscles when running near-to-MSS [31]. Exposures at D-1 may not allow those muscles to be completely recovered on match day, and the stimulus (short-term conditioning effect?) may fade away when performed too early in the week (D-3 and earlier), losing its ‘protective effect’. Clearly, studies examining this recovery times course in ecological conditions would help better understand this programming aspect. Practically, if D-2 was to be the most appropriate day for near-to-MSS exposures as per the current results (Figure 3 and 4), the programming of the other days of the week may need to be tailored accordingly (i.e., D-4 and D-3), so that players do not reach D-2 with excessive levels of neuromuscular fatigue – not to be at higher risk of hamstring injuries during the exposures themselves. Additionally, while those D-2 exposures may concern the entire squad for long turnarounds (i.e., 6- to 8-d), they may only concern subs for 3- to 5-d turnarounds. In this latter scenario, practitioners reported programming these exposures either on match day immediately post-match, at D+1 or D+2 (in relation to potential days off).^12^ The present D-2 practice is then straightforward when that day is either a D+1 (3-d turnaround), or a D+3 (4-d turnaround). For 5-day turnarounds, the option could be to delay this exposure up to D+3/D-2, and/or spread it across multiple days (match day and then again D+3/D-2). As always, players and practitioners’ experiences would dictate the possible applications of the present findings in their own context [32].

The lack of clearer differences between the different exposures scenarios (CIs overlap) – despite the very large data set – is likely related to the fact that other factors than the programming of near-to-MSS exposures *per se* may have a greater effect on injury rate, and, in turn, could have diluted/confounded the univariate analysis. This is an important limitation of the present analysis. While we thought to answer the simple question of the programming of near-to-MSS exposures, it is clear that injuries are largely multifactorial in nature [33] and that different chronic training loads and match minutes prior to the turnarounds examined, may also directly affect injury rates. However, we deliberately decided to zoom within each turnaround, since this is the way the very large majority of practitioners operate in the football field, taking and programming one turnaround after the other, with each of them being almost independent of the previous [12]. Additionally, the simultaneous consideration of player profiles (e.g., age, injury history, strength, mobility or flexibility) and other measures of internal training load and responses to load should also improve the analysis – while making the current outputs less straightforward for practitioners. There is in fact a trade-off between the desire for simple questions to have simple answers (e.g., when is it best to sprint?) and more sophisticated analytic approaches that may have more precision but require more effort to interpret in order to provide direct applications (i.e., results of multivariate analyses can be difficult to translate into simple yes/no answers).

### Limitations

In the absence of consistent MSS testing practices across the different teams examined, players’ MSS was determined from the available training- and match-related GPS data. While recent results have shown that players may be able to reach their true MSS during matches and some specific training sessions [20], we were not able to verify this at the individual player level. It is therefore possible that inaccurate MSS were used in the analysis, which may have added noise to the results. At least the same (GPS) technology was used across all teams to effectively measure MSS. Importantly, we chose to express injury rate / 1000 turnarounds and not / exposure as it is traditionally done [1, 2]. While it is more intuitive to do so (i.e., exposure to very high-speed running likely is related to the number of hours of training exposure a coach will program), the use of 1000 turnarounds was preferred for the following reasons: 1) exposure duration was not consistently reported across the team, i.e., different ways to report sessions duration (based either on GPS data that may be split differently between practitioners vs. manual entries in teams calendars), and 2) a low number of exposures (e.g., especially for 3-d or 4-d turnarounds; even more when a day off is programmed) can lead to wide variance in the reported rates (potentially very low or very high) with point estimates that are unrepresentative of the true injury rates. Another potential limitation is related to the data used, and the teams it was drawn from i.e., users of the Kitman Labs platform. It is possible that these teams are particularly inclined to embrace evidence-informed practices, and as a result are biased toward the importance of near-to-maximal bouts exposures (as per the literature [8–10]). It is impossible to assess to what extent this is a legitimate concern, but our view is that the cross-section of teams from different leagues across the globe over a number of years makes this less of a concern. Finally, the injury records used for analysis are as good as what practitioners may have registered. Relying on injuries based on practitioners’ entries is however common practice [1], and we believe that the value of the information provided, derived from a very large sample size (n = 24486 player-turnarounds), partly outweighs those possible limitations. We will certainly continue to investigate this, and other topics related to planning the microcycle but given the lack of research in this area, we encourage other researchers to think about experimental designs which would provide more insight on how best to adapt the training schedule to the fixture schedule.

### Practical applications

While the present observational study design precludes the examination of causal relationships, reaching > 95% of MSS during training may be more protective against non-contact time loss match hamstring injuries than not reaching at least 85% of MSS, or only > 85% or > 90% of MSS. Additionally, programming 95% exposures at D-2 may be the more relevant strategy to decrease the incidence of non-contact match hamstring injuries than no programming exposures at all, or having those exposures at D-3 and/or earlier in the week. If D-2 was to be the most appropriate day for near-to-MSS exposures, the programming of the other days of the week needs to be tailored accordingly (i.e., D-4 and D-3), so that players do not reach D-2 with excessive levels of neuromuscular fatigue – in order not to be at higher risk of hamstring injuries during the exposures themselves.

## CONCLUSIONS

Using a very large data set (for a total of 620 players across 5052 training sessions and 36 team-seasons), we showed for the first time that the large majority of players arrived at the match without having been exposed to near-to-MSS running bouts during the training days of the current turnaround. However, while association does not imply causation, match hamstring injuries in elite football were systematically lower when > 95% MSS exposures were programmed at D-2. The present results should nevertheless be taken with caution, and the possibility of false positives should not be disregarded. Future replication studies are required to confirm the present findings on different samples. Whether the current question could one day be answered ecologically using a randomized trial design looks complex at the moment, but this would definitely improve our confidence in the training programming strategy suggested by the present findings.
